# Leprosy Reactions Show Increased Th17 Cell Activity and Reduced FOXP3+ Tregs with Concomitant Decrease in TGF-β and Increase in IL-6

**DOI:** 10.1371/journal.pntd.0004592

**Published:** 2016-04-01

**Authors:** Chaman Saini, Anisuddin Siddiqui, Venkatesh Ramesh, Indira Nath

**Affiliations:** 1 National Institute of Pathology (ICMR) Safdarjung Hospital Campus, New Delhi, India; 2 Government Autonomous Holkar Science College, Indore, India; 3 Department of Dermatology, Safdarjung Hospital New Delhi, India; Fondation Raoul Follereau, FRANCE

## Abstract

**Background:**

50% of leprosy patients suffer from episodes of Type 1/ reversal reactions (RR) and Type 2/ Erythema Nodosum Leprosum (ENL) reactions which lead to morbidity and nerve damage. CD4^+^ subsets of Th17 cells and CD25^+^FOXP3^+^ regulatory T cells (Tregs) have been shown to play a major role in disease associated immunopathology and in stable leprosy as reported by us and others. The aim of our study was to analyze their role in leprosy reactions.

**Methodology and Principle Findings:**

Quantitative reverse transcribed PCR (qPCR), flowcytometry and ELISA were used to respectively investigate gene expression, cell phenotypes and supernatant levels of cytokines in antigen stimulated PBMC cultures in patients with stable disease and those undergoing leprosy reactions. Both types of reactions are associated with significant increase of Th17 cells and associated cytokines IL-17A, IL-17F, IL-21, IL-23 and chemokines CCL20, CCL22 as compared to matching stable forms of leprosy. Concurrently patients in reactions show reduction in FOXP3^+^ Treg cells as well as reduction in TGF-β and increase in IL-6. Moreover, expression of many T cell markers, cytokines, chemokines and signaling factors were observed to be increased in RR as compared to ENL reaction patients.

**Conclusions:**

Patients with leprosy reactions show an imbalance in Th17 and Treg populations. The reduction in Treg suppressor activity is associated withhigherTh17cell activity. The combined effect of reduced TGF-β and enhanced IL-6, IL-21 cytokines influence the balance between Th17 or Treg cells in leprosy reactions as reported in the murine models and autoimmune diseases. The increase in Th17 cell associated cytokines may contribute to lesional inflammation.

## Author Summary

Reversal reactions (RR; Type 1) and Erythema Nodosum Leprosum (ENL; Type 2) are two types of leprosy reactions which appear episodically in a proportion of leprosy patients and lead to high morbidity and peripheral nerve damage that require immediate medical attention, ENL seen in anergic lepromatous patients, show immune complexes as well as transient emergence of T cell functions. Lesions of RR with borderline tuberculoid background show pathological features associated with delayed-type hypersensitivity. The present study shows increased Th17 cell show activity in reaction patients as compared to the matching stable leprosy type and which may contribute to the inflammation/immunopathology observed in the lesions. This increase is accompanied by reduction in Treg cells and their inhibitory activity. The balance between Th17 and Treg cells may be influenced by the combined effects of reduction in TGF-β and increase in IL-6, IL-21 cytokines. Of interest was the expression of more genes associated with T cells, cytokines, chemokines, signaling and transcription factors by RR as compared to ENL patient.

## Introduction

Leprosy reactions occurring in approximately 50% of leprosy patients cause severe morbidity and need immediate clinical attention. Whereas the stable leprosy forms run a bland course amenable to multi drug therapy, leprosy reactions can be triggered by treatment and can also occur after the completion of treatment. Leprosy is a chronic infection of skin and peripheral nerves caused by *Mycobacterium leprae* and is of public health concern in India, South America, Central Africa and South East Asia. The global prevalence of leprosy was reported by WHO to be 180, 618 cases in 2014, while the number of new cases reported in the same year was 215,656[1]. Research has been centered around the diverse clinico-pathological presentation of leprosy in man [2], where the polar forms tend to remain stable whereas the borderline forms are vulnerable to reactions and morbidity. Tuberculoid leprosy presents as both polar (TT) and borderline (BT) forms with well defined an aesthetic skin patches which are paucibacillary and prone to early peripheral nerve damage. In contrast, the lepromatous forms of polar (LL) and borderline (BL) forms show diffuse involvement of skin and other organs with presence of varying load of the pathogen in macrophages, endothelial and Schwann cells. Leprosy reactions are mainly of 2 types, Type 1 or reversal reactions (RR) are seen in borderline leprosy forms of BT, BB and BL where there is inflammation localized to the dermal patch and the neighboring peripheral nerve[3]. Acute neuritis is painful and is a major medical emergency which when unattended leads to nerve damage and deformity. On the other hand, Type 2 reaction specially ENL, appears in BL/LL patients who show systemic features accompanied by fever, joint pains and small reddish nodules scattered over the body along with peripheral nerve involvement. Patients at the tuberculoid and lepromatous poles show reverse patterns in cell mediated immunity and antibody responses to the *M*.*leprae* antigens and have been reported to be associated respectively with Th1, Th2 paradigm[4] with some patients showing Th0 profile[5]. The immune mechanisms underlying the exquisite antigen unresponsiveness in lepromatous leprosy and the leprosy reactions are yet to be fully understood. Leprosy reactions specially ENL have been shown to be associated with enhanced T cell activity, altered cytokine pattern and a Th1 shift[6]. Immune complexes were seen both in the serum and tissues of ENL patients[7]. The triggering factors in the inductions of these reactions are not known although motifs of LSR2protein of *M*.*leprae* have been shown to be recognized during ENL[8].

In addition to the conventional CD4^+^ subsets of Th1 and Th2 cells, additional subsets of Th17 and CD25^+^FOXP3^+^Tregulatory cells were discovered initially in models of autoimmunity[9] followed subsequently in various human diseases including autoimmune states[10] and infectious diseases caused by mycobacteria [11], respiratory syncitial virus [12] and HIV [13]. CD4^+^ Th17 cells have an important role in autoimmune inflammation, clearance of pathogens and tissue pathology [14,15,16]. They produce IL-17A, IL-17F and IL-22 which lead to tissue inflammation and destruction [17,18]. On the other hand Treg cells have a suppressor/inhibitory role and regulate inflammation[19,20] and maintain tolerance[9]. The differentiation pathways of these two subsets are influenced by various cytokines. TGF-β is essential for differentiating naive T cells towards Th17 cells[21]. It acts in concert with the pro inflammatory cytokine IL-6 to induce the transcription factor RORC that leads to Th17 differentiation. Treg cells appear to retard the differentiation of Th17 cells [22] where relative levels of TGF-β and IL-6 determine the outcome [23,24].

Our and other studies had shown that CD4^+^FOXP3^+^T regulatory cells producing TGF-β were increased in stable lepromatous patients which may explain the anergy associated with this leprosy type [25,26]. Moreover we reported that Th17 cells may constitute the third subset of Th cells in leprosy patients who failed to show Th1 and Th2 polarization[27]. Others reported IL-17F increase may be an early marker of the reversal reactions[28]. The present study is aimed at understanding the mechanisms that lead to immune inflammation and immune-regulation during leprosy reactions. We investigated patients suffering from both RR and ENL reactions and compared them with matching non reactive stable forms of BT and LL respectively. *M*.*leprae* (MLSA) stimulated PBMC from patients were investigated for gene expression by qPCR, flowcytometry for phenotype identity and ELISA for cytokine levels in culture supernatants.

## Materials and Methods

### Ethics statement

Informed written consent for blood and skin biopsies was obtained from patients following approval of the study by the Institutional Ethical Committee [08-09-EC (3/7)] of Safdarjung Hospital, New Delhi, India.

### Patients

Sixty six newly diagnosed leprosy patients (44 males, 22 females aged between 19–60 years) attending the Leprosy Clinics of the Department of Dermatology, Safdarjung Hospital, New Delhi were included in the study (Table 1). Leprosy type was determined on the basis of clinical and histological criteria as per the Ridley-Jopling classification [2,29]. The study group included 30 leprosy patients in reactions prior to institution of anti-reaction therapy: 15 each were in type 1/reversal reactions (RR) and type 2/Erythema Nodosum Leprosum (ENL); 36 freshly diagnosed patients with stable leprosy without previous history or clinical evidence of reactions were included as controls and consisted of 18 each of borderline tuberculoid (BT) and lepromatous (LL) leprosy. Exclusion criteria included patients below 18 years of age, pregnancy, clinical evidence of anemia and other infections such as tuberculosis, HIV and helminthic infestation. In this study antigen (MLSA) stimulated and unstimulated PBMC were investigated for i) gene expression in 10 BT, 9 BT with RR 10 LL, and 9 LL with ENL reactions ii) T cell phenotypes were identified with flow cytometry in 8 each of BT and LL subjects and 6 each of BT-RR and LL-ENL patients and iii) ELISA on selected cytokines was undertaken on PBMC culture supernatants of all four clinical groups.

**Table 1:** Clinical details of 66 newly diagnosed untreated leprosy patients.

Leprosy Type	Patients	Age	Sex		Duration of disease	Duration of reaction	BI
	No	Range	M	F	(months)	(days)	
BT	18	19–48	11	7	2–24	-	0–0.5
BT-RR	15	22–48	10	5	1–24	8–180	0–1.6
LL	18	24–60	12	6	6–15	-	5–6
LL-ENL	15	22–60	11	4	3–36	8–28	1.2–5.5
Total	66	19–60	44	22	1–36	8–180	0–6

Patients were typed on the basis of Ridley Jopling classification [2,29], BI; Bacillary Index (mean of six lesional sites), M; male, F; female. Borderline Tuberculoid leprosy; (BT), Borderline Tuberculoid Reversal Reaction; (BT-RR), Lepromatous Leprosy; (LL), Erythema Nodosum Leprosum; (ENL), Bacterial Index; (BI)

### Isolation of peripheral blood mononuclear cells (PBMC)

10 ml of venous blood was collected in heparinized sterile tubes (10 units/ml). PBMC were separated by density gradient centrifugation at 800g for 20 min on Ficoll-Hypaque (Histopaque, Sigma Aldrich, USA) as described earlier [27]. Cells were washed three times in sterile 1x HBSS (GIBCO NY, USA) and re-suspended as described below. Cell viability by 0.2% trypan blue staining (Sigma Aldrich, MO, USA) ranged from 95–98%.

### *Ex vivo s*timulation of PBMC cultures

*Ex vivo* PBMC cultures were undertaken as described previously [27]. In brief, 1.5x10^6^ cells /ml suspended in RPMI 1640 (GIBCO NY, USA) with 10% pooled human AB serum, 2mM L-glutamine, 100 units of penicillin (Sigma Aldrich, MO, USA) and 100 μg streptomycin (Sigma Aldrich, MO, USA) were cultured in sterile flat bottom 24- well plates (Falcon, USA) as follows: cells were stimulated with and without 10 μg/ml of *M*. *leprae* sonicated antigen (MLSA) kindly provided by Dr. P J Brennan(Colorado State University, USA). Cultures were optimized for incubation in earlier studies [27] to 48 h at 37°C in humidified 5% CO_2_ + air. After incubation, harvested cells were washed as above and stored in RNA later (Sigma Aldrich, MO, USA) for gene expression studies or processed for flow cytometry analysis as given below.

### RNA isolation and reverse transcriptase PCR reaction

RNA was isolated from cultured PBMC and skin lesions using RNeasy Mini Kit (Qiagen, Maryland, USA) according to the manufacturer’s instructions. The isolated RNA was quantified using nanodrop spectrophotometer (Nanodrop Technologies, Wilmington, USA) and purity at 260/280 from 1.8 to 2.0 was considered to be optimum. The quality of RNA was also checked for 28s and 18s RNA by electropherogram using Bio analyzer (Agilent Technologies, Inc., Singapore). RNA Integration Number value of >7 was considered to be optimum. For cDNA synthesis 1 μg total RNA was transcribed with RT First strand kit (SA Biosciences, MD, USA). Reactions were performed as per the manufacturer’s instructions and the cDNA stored at -20°C till further use.

### PCR array

This study employed a commercially customized PCR array for 84 genes (accession numbers of genes and primers were provided by PAHS 073, SA Biosciences, Qiagen Co. CA,USA, (S1 Table) and used as per the manufacturer’s instructions and included primers for expression of genes for cell surface markers- CD28, CD34, CD3D, CD3E, CD3G, CD4, CD40LG, CD8, ICAM1, ICOS, ISG20, cytokines- CSF2, CSF3, IFN-γ, IL-10, IL-12B, IL-13, IL-15, IL-17A, IL-17C, IL-17D, IL-17F, IL-18, IL-1β, IL-2, IL-21, IL-22, IL-23A, IL-25, IL-27, IL-3, IL-4, IL-5, IL-6, TGF-β1, TNF, cytokine receptors-IL12RB1, IL12RB2, IL17RB, IL17RC, IL17RD, IL17RE, IL23R, IL6R, IL7R, chemokines- CCL1, CCL2, CCL20, CCL22, CCL7, CD247, CX3CL1, CXCL1, CXCL12, CXCL2, CXCL5, CXCL6, IL8, MMP13, MMP3, MMP9 signaling and transcription markers- CACYBP, CEBPB, CLEC7A, S1PR1, FOXP3, GATA3, JAK1, JAK2, NFATC2, NFKB1, RORC, SOCS1, SOCS3, STAT3, STAT4, STAT5A, STAT6, SYK, TBX21, TIRAP, TLR4, TRAF6, YY1: Housekeeping genes consisted of β2M, HPRT1, RPL13A, GAPDH, ACTB. It was ensured that the cDNA used had optical density (OD) >1.7 at 260/280 wavelength. 1 μg of cDNA was used per reaction/ well containing the ready to use PCR master mix and appropriate primers. These were then subjected for 2 h to quantitative reverse transcribed PCR (qPCR, ABI 7000, Applied Biosystems Singapore)). Threshold cycle values were normalized and expressed as ΔCt: mean Ct of gene of interest-mean Ct of set of 5 housekeeping genes. Fold change in gene expression was calculated by 2-ΔΔCt method using manufacturer’s software. Heat maps showing level of expression of genes were visualized as magnitude/intensity of fluorescence using online software (pcrdataanalysis.sabiosciences.com) provided by SABiosciences, Qiagen. Patients with RR were compared with BT and ENL with LL.

### Flowcytometry analysis of cell surface and intracellular markers of PBMC cultures

All reagents were obtained from BD Biosciences, San Diego, CA and used as per manufacturer’s instructions as described earlier[25,27]. 8 h prior to harvest, *ex vivo* cultured cells were incubated with monensin (BD Golgi Stop) to block secretion of cytokine. For surface staining, 0.5 x 10^6^cells/50 μl in staining buffer were incubated with a cocktail of anti human CD3 (Per-Cpcy-5.5, clone UCHT1), CD4 (APC-H7, Clone SK3), CD25 (FITC, clone M-A251) and CCR6 (PE-Cy7 clone) with respective isotype controls. After the surface staining, cells were washed two times and permeabilized with permeabilizing / fixation solution (containing saponin/paraformaldehyde) for 30 min at 4°C. The cells were washed as above and resuspended in Perm/Wash buffer and incubated with anti human IL-17A (Alexa Fluor-647, clone 64DEC17), IL-17F (Alexa Fluor-488, clone O33-782), IL-6 (PE, clone MQ2-6A3) and IL-21 (PE, clone-3A3-N2.1) at 4°C for 30 min in the dark followed by two washes as before, resuspended in 500 μl. For nuclear FOXP3 staining, cells were incubated with 1xFOXP3 buffer A for 10 min at room temperature; cells were washed as above and permeabilized with buffer C for 30 min at room temperature. The cells were washed as before, resuspended in stain buffer and incubated with anti- FOXP3 (APC, clone 259D/c7) and anti TGF-β (PE, clone TW4-9E7) at room temperature for 30 min in the dark followed by two washes as before and suspended in 500μl of staining buffer. To detect phosphorylation status of STAT3, cultured cells were first fixed for 10 min at room temperature, permeabilized as before with appropriate buffer and stained with a cocktail of PE labeled anti mouse pSTAT3, anti human IL-17A, CD3, CD4 and CCR6 antibodies. Stained cells were acquired by FACS aria (BD Biosciences, San Diego, CA) and analyzed by BD FACS Diva software.

### Estimation of cytokines by ELISA

Cytokines (IL-17A/F, IL-21, IL-22, IL-23A, IL-6, IL-1β, IFN-γ and TGF-β) were measured in duplicate by ELISA in culture supernatants from unstimulated and antigen stimulated PBMCs of leprosy patients (Ready Set Go, e-Bioscience, San Diego, CA, USA) as per manufacturer’s instructions. In brief, 96-well plates (Nunc, Rochester, NY, USA) were coated overnight at 4°C with biotin conjugated anti human antibodies for each of the cytokines, Plates were washed 5 times, blotted and wells blocked with assay diluents for 1 h at room temperature. 100ul/well of culture supernatant was added and plates incubated overnight at 4°C. After washing with buffer, avidin-horseradish peroxidase-conjugated anti-mouse antibody was added and the plates incubated at room temperature for 30 min. Subsequent to washing wells as before, color development was undertaken using TMB (Tetramethylbanzedine) substrate and the reaction stopped by 1 N H_2_SO_4_. The optical density (OD) of each well was read at 450 nm.

### Statistical analysis

Graph Pad Prism version 5 (GraphPad Software, Inc., San Diego, CA, USA) was used for two tailed Mann-Whitney for significance and Spearman nonparametric correlation coefficient. Student t test was used for fold change differences as per the manufacturer’s software. p< 0.05 was considered as statistically significant. Heat map of gene expression were established by online software of SA bioscience (pcrdataanalysis.sabiosciences.com).

## Results

*Ex vivo* antigen stimulated and unstimulated PBMC were investigated in patients with Type 1/reversal reaction (RR) and Type 2/ erythema nodosum leprosum (ENL) leprosy reactions prior to institution of anti reaction therapy with steroids, clofazamine or thalidomide. They were compared with complementary clinical groups of BT or LL respectively where the subjects were freshly diagnosed, untreated and without clinical evidence or previous history of reactions. Quantitative real time PCR (qPCR) was used for gene expression, flow cytometry for phenotypic characterization and intracellular cytokines and ELISA for measuring cytokines in culture supernatants. The demographic details of the 4 clinical groups of leprosy patients are given in Table 1.

### Th17 cell increase in leprosy reactions

#### qPCR

**Fig 1:**
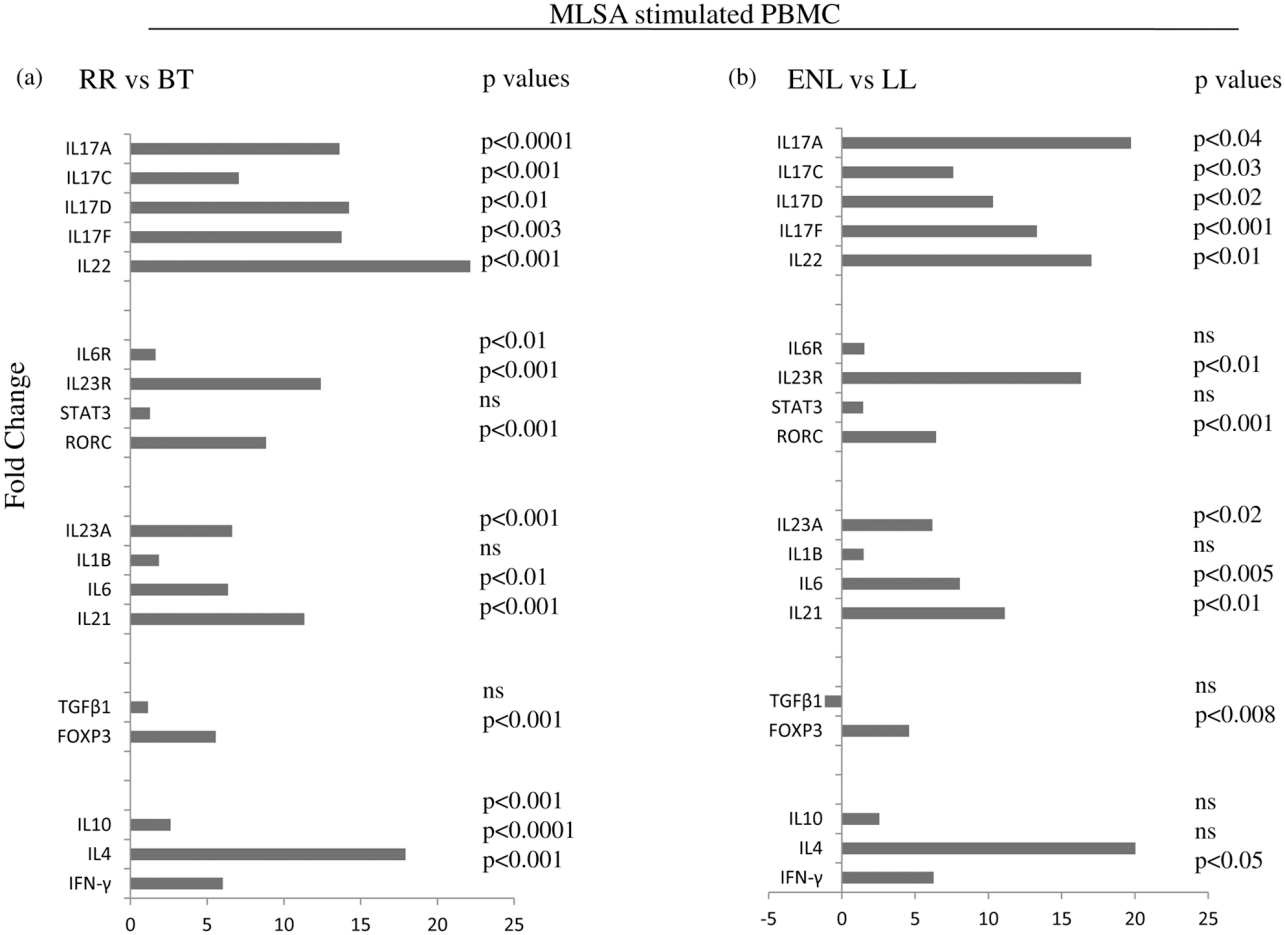
Fold change in gene expression of Th17, Treg, Th1 and 2 associated markers in leprosy reaction patients (RR,ENL) as compared to matching stable leprosy patients (BT, LL) as evaluated by qPCR of *ex-vivo* antigen (MLSA) stimulated PBMC. Horizontal histogram shows significant fold increase in (a) RR, (b) ENL as compared to BT, LL patients respectively for gene expression of Th17 and Treg associated cytokines, receptors and transcription factors as well as Th1 and Th2 cytokines. p value was calculated based on a Student’s t-test and p< 0.05 was considered to be statistically significant. Abbreviations: BT; borderline tuberculoid, RR; reversal reaction, LL; lepromatous leprosy, ENL; erythema nodosum leprosum as per Ridley Jopling classification[2,29], ns = non significant.

Fig 1 shows a significant increase in fold changes in RR group as compared to stable BT patients for all IL-17 isomers in MLSA stimulated PBMC (IL-17A p< 0.0001, IL-17F p<0.003, IL-17C p<0.001, IL-17D p<0.01; the respective fold change was 13.6, 13.7, 7.0 and 14.2. ENL patients also showed significant but relatively higher increase in fold changes ranging from 7.6 to 19.7 (p<0.02–0.001) as compared to stable LL group (Fig 1b). Of interest was the greater fold change in IL-17A as compared to other IL-17 isomers. The increased fold change seen in ENL as compared to RR is mainly due to the low baseline levels noted in the anergic LL background type of leprosy (Table 2). RORC the molecular signature of Th17 cells also showed fold increase in both reactions (p<0.001) with relatively higher fold change in RR (8.8) as compared to ENL (6.4). The above features were further confirmed by ELISA in PBMC culture supernatants of leprosy patients with and without reactions (Table 3). There was significantly higher (p<0.0001) levels of IL-17A/F in RR and ENL patients as compared respectively to non reactive BT and LL. Stable LL patients showed the lowest levels of cytokines as compared to other clinical types as reported earlier [27].

**Table 2:** Mean ΔCt ±SD of cytokines, signaling molecules, and transcription factors of 48 hr. MLSA stimulated PBMC from stable (BT, LL) and reactions (RR, ENL) leprosy patients.

	ΔCt (Mean±SD)				P value		
Gene	BT	LL	ENL	RR	BT vs RR	LL vs ENL	RR vs ENL
**IL-17A**	6.6±2.5	12.9±2.4	5.5±3.3	4.1±0.6	0.0003	0.002	ns
**IL-17C**	6.1±1.5	9.1±2.0	5.3±2.6	4.5±0.7	0.006	0.005	ns
**IL-17D**	8.6±0.5	11.9±1.6	7.8±3.1	6.5±0.8	0.0002	0.01	ns
**IL-17F**	5.2±0.4	9.6±2.3	4.3±1.9	3.8±0.8	0.001	0.001	ns
**IL-22**	5.4±2.2	10.0±2.8	4.6±3.3	2.9±0.5	0.0003	0.004	ns
**TNF-α**	4.3±0.8	4.7±0.8	5.1±1.3	4.0±0.9	ns	ns	0.01
**IL-6R**	3.7±0.6	3.1±0.6	3.5±0.7	3.2±1.4	ns	ns	ns
**IL23R**	5.0±0.8	10.2±3.2	4.7±3.6	3.5±0.8	0.004	0.006	ns
**STAT3**	2.9±0.7	3.0±0.3	2.2±0.9	2.5±0.6	ns	ns	ns
**FOXP3**	7.2±1.8	5.0±1.6	5.5±1.7	4.8±0.8	0.001	ns	ns
**RORC**	5.2±0.5	8.8±0.8	5.0±2.6	3.5±0.8	0.001	0.004	ns
**IL-23A**	4.6±0.7	7.2±1.0	3.8±2.0	3.1±0.5	0.0004	0.004	ns
**IL-1β**	2.3±1.3	3.1±1.3	3.0±1.3	1.9±1.0	ns	ns	ns
**TGF-β**	3.2±0.5	2.4±0.7	3.0±1.0	2.7±1.1	ns	ns	ns
**IL-6**	5.0±0.8	7.3±2.8	4.4±2.0	3.6±0.6	0.001	0.04	ns
**IL-21**	4.7±0.5	9.0±2.3	5.3±3.5	3.3±0.7	0.002	0.03	ns
**CCL20**	5.5±1.5	6.6±2.4	4.0±1.8	3.3±0.7	0.001	0.02	ns
**CCL22**	4.2±1.2	5.0±1.2	3.6±0.9	3.2±0.9	ns	0.02	ns

Classification of patients according to Ridley Jopling[2,29] as in legend to Table 1. Peripheral blood mononuclear cells (PBMC) *p< 0.01, ** p< 0.001, *** p< 0.0001 by two tailed Mann Whitney test. P<0.05 was considered significant, ns = non significant

**Table 3:** Mean pg/ml ± SD of cytokines in culture supernatants of 48 hr. *M*.*leprae* stimulated PBMC from 10 each of stable tuberculoid (BT), reversal reaction (RR) stable lepromatous leprosy (LL) and erythema leprosum reaction (ENL) patients investigated for gene expression study.

Cytokine	BT	RR	LL	ENL
IL-17A	101.9±2.6	219.8±28.07***	45.52±22.7	650±195.2***
IL-21	314±19.1	346±30.4*	267.4±11.5	232.5±48.5
IL-22	561±118	492.8±115.0	633.1±89.1***	96.72±93.0
IL-23A	62.6±51.2	131.9±45.9**	23.64±10.4	48.51±45.2*
IL-1β	13.6±16.3	7.3±1.1	8.5±4.4	22.4±12.2**
IL-6	50.5±27.9	122±21.6***	21.8±10.9	439.1±203.0**
TGF-β	104.4±45.7	80.5±13.0	312±98.1	297.5±65.8

Classification according to Ridley Jopling classification [2,29]. Legend as in Table 2

*p< 0.01

** p< 0.001

*** p< 0.0001 by two tailed Mann Whitney test. P<0.05 was considered significant.

#### Flowcytometry

**Fig 2:**
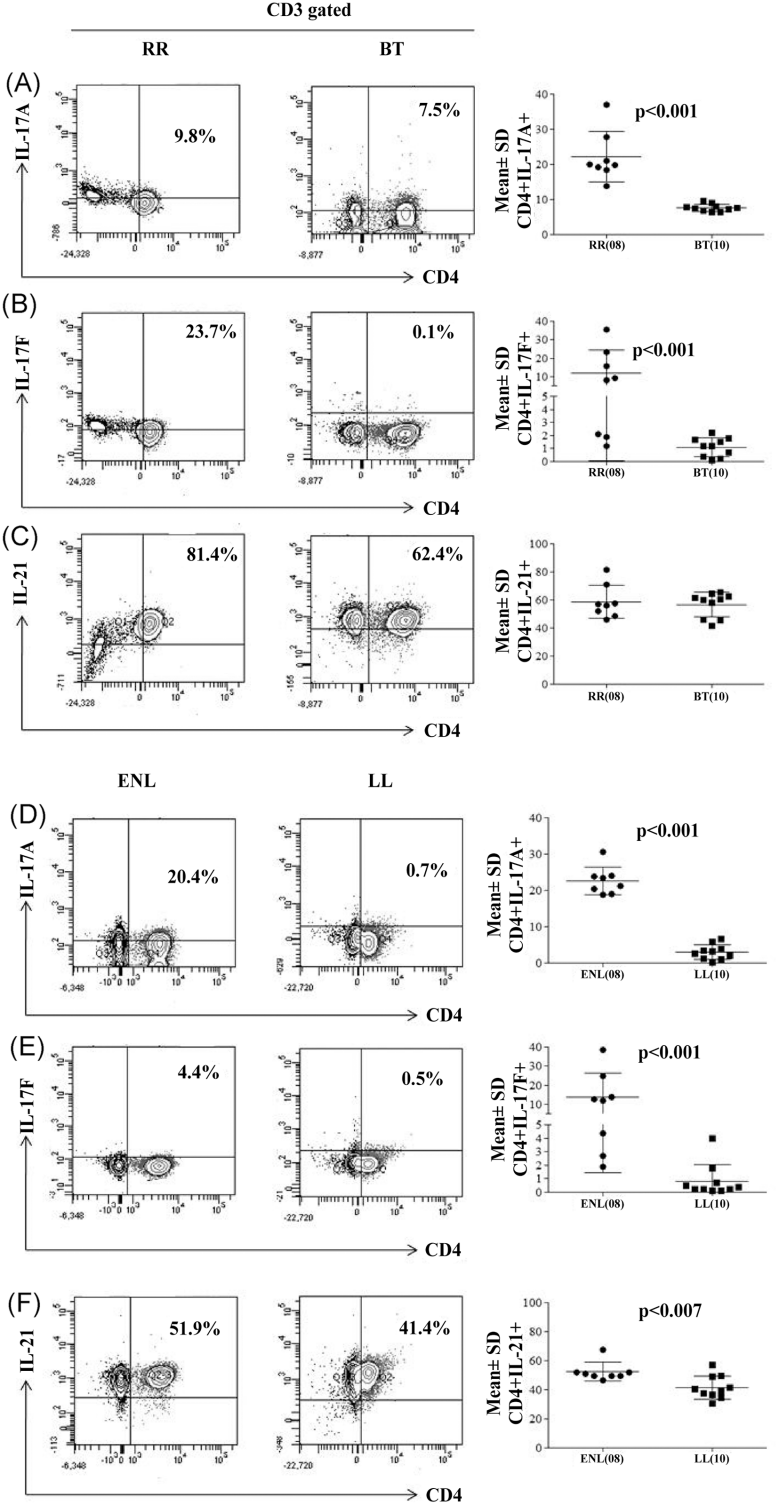
Increase in IL-17 and IL-21 bearing CD3^+^CD4^+^ T cells in patients undergoing leprosy reactions. Flowcytometry analysis of antigen stimulated PBMC from one each of representative patient with RR, BT, ENL, and LL leprosy (see Materials and Methods). Dot plot shows percentage of CD3^+^ gated CD4^+^ cells with intracellular IL-17A (Figs A, D), IL-17F (Figs B, E) and IL-21 (Figs C, F) in BT, RR and LL, ENL subjects. Scattergram (Mean % ± SD of cells) confirms the significant increase in percentage of Th17 cells in RR, ENL compared to BT, LL patients respectively for IL-17A, IL-17F cytokines as indicated by p values. IL-21^+^ cells were increased only in ENL. p< 0.05 was considered statistically significant (two tailed Mann-Whitney). (), parenthesis shows number of subjects tested. Abbreviations as in Fig 1.

Further validation of the above data was obtained with flow cytometric analysis to identify the T cell phenotype in 48 h MLSA stimulated PBMC cultures. As expected (Fig 2), the intracellular presence of IL-17A, F and IL-21 was associated with CD3^+^CD4^+^ cells in all the clinical groups. Reversal reaction (RR) patients showed significantly higher (p<0.001) percentages of CD3 gated CD4^+^IL-17A^+^ and CD4^+^IL-17F^+^ cells as compared to non-reactive BT subjects (Fig 2A and 2B). However, there was no difference in the percentage of IL-21^+^cells in RR and stable BT groups (Fig 2C).

ENL patients (Fig 2) also showed significant (p<0.001) increases in the percentages of IL-17A^+^ (Fig 2D), IL-17F^+^ (Fig 2E) cells similar to patients with reversal reactions. Unlike RR patients with ENL showed an increase in IL-21^+^ cells (Fig 2F) as compared to stable non- reactive LL patients. In conclusion, Th17 cells with intracellular IL-17A, F are increased in both types of leprosy reactions whereas CD4^+^IL-21^+^ cells were higher only in ENL reactions patients.

### Other Th17 associated cytokines, receptors and chemokines in *ex vivo* 48hr PBMC cultures

Cytokines IL-1β, IL-23 which have been reported to influence and sustain Th17 lineage [30,31] were investigated (Fig 1). In general, the expression of IL-23A was significantly up regulated in *ex vivo* antigen stimulated PBMC of reactions patients compared to non reactive patients reflecting differences due to recall responses in antigen stimulated PBMC as compared to the status of an ongoing *in vivo* response. Fold change of IL-23Aexpression was significantly up regulated in both RR(p<0.001) and ENL(p<0.02,) as compared to BT and LL patients respectively. Moreover, cytokine levels in *ex-vivo* cultures by ELISA supported data obtained with qPCR. (Table 3)

IL-23R expression also showed significant fold change/increase in RR and ENL, with the latter showing a higher increase in RR (p<0.001) as compared to ENL (p<0.01). Expression of IL-23R also correlated with their expression of respective ligands (IL-23A) as indicated by Spearman r correlation of (r^2^ = 0.92, p<0.0001 and r^2^ = 0.71, p<0.0001) respectively indicative of their functional relevance. IL-1β expression did not show any difference in RR and BT patients (Fig 1 and Table 3). However, at protein level IL-1β showed a preferential increase in ENL by ELISA indicating that 48h culture period may have missed the time kinetics of gene expression whereas supernatants reflected the accumulation of the translated product.

Since leprosy reactions are inflammatory in nature we also looked at the role of common chemokines and cytokines. As shown in Table 2, expression of CCL20, and CCL22 were seen to be highest in antigen stimulated PBMC cultures of RR and ENL as compared to stable disease. Both of these chemokines showed significant increases in fold changes in RR and ENL (p<0.001, p<0.02) as compared to BT and LL respectively. CCL22 showed increased fold change only in ENL (p<0.02) and not in RR patients. Cytokines associated with Th1 and Th2 subsets showed fold change increases in both reactions. Whereas RR showed increase in both Th1[32] and Th2 cytokines, ENL showed significant fold change increase only in IFN-γ as reported earlier[8].

### CCR6^+^Th17 cell increase in leprosy reactions

**Fig 3:**
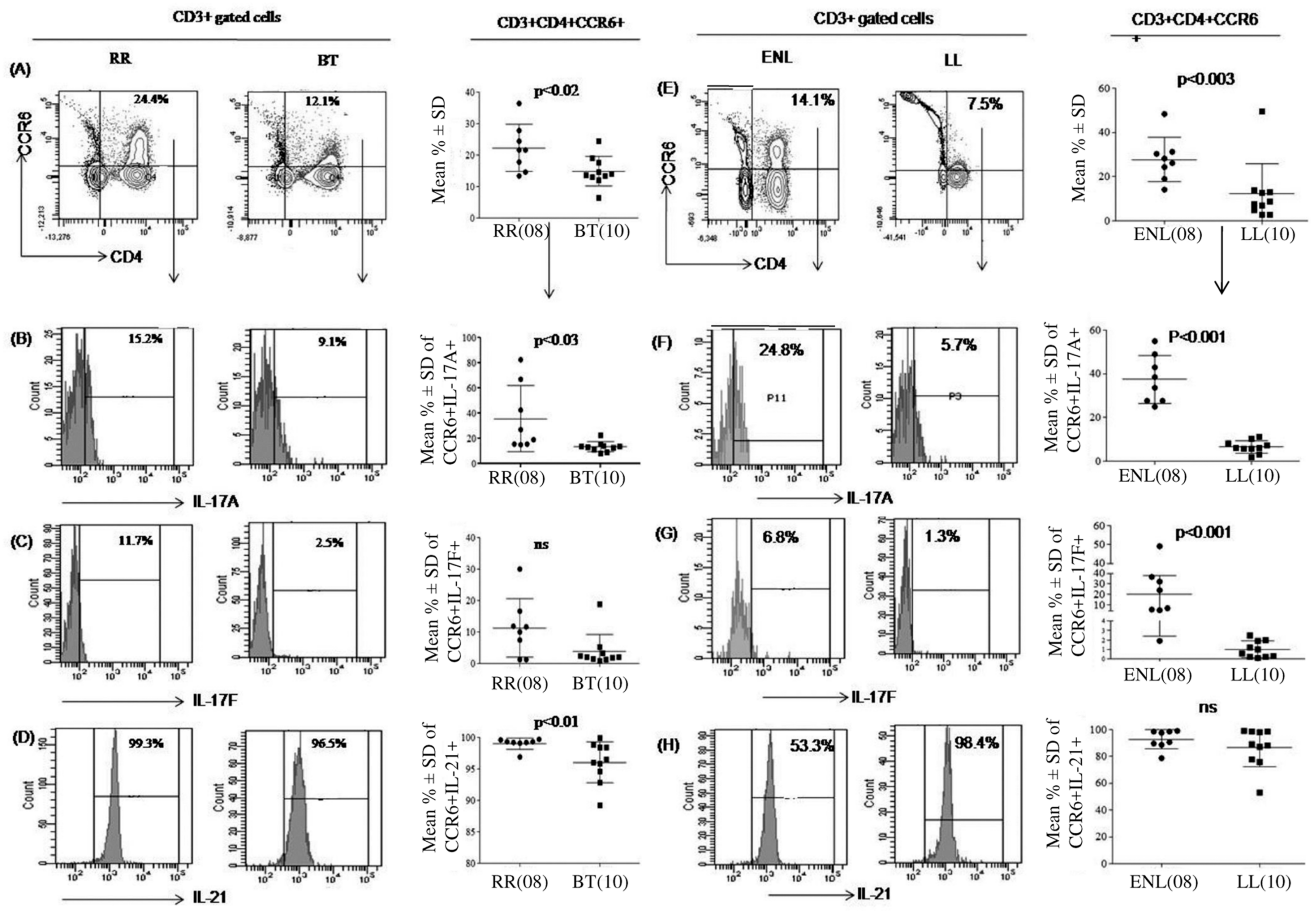
CD4+CCR6+ effector Th17 cells are increased in patients undergoing leprosy reactions. Flowcytometry analysis of antigen stimulated PBMC from one representative patient of RR, BT, ENL and LL (see Materials and Methods). Fig 3 and e show dot blots with increase in percentage of CD3^+^ CD4^+^CCR6^+^ cells in RR as compared to BT (A) and ENL as compared to LL patient(E). Histograms show increase in percentages of intracellular IL-17A^+^ (B, F), IL-17F^+^ (C, G) and IL-21^+^ cells (D, H) in both leprosy reactions as compared to stable leprosy patients. Scattergrams show individual data with Mean % ± SD of IL-17A/F, IL-21 in patient groups. p< 0.05 was considered statistically significant (two tailed Mann-Whitney). (), Parenthesis shows number of subjects tested. Abbreviations as in Fig 1.

We next investigated the expression of CD4^+^IL-17^+^ cells with CCR6, an effector /memory T cell marker [33] using flow cytometry. During both RR (Fig 3A) and ENL (Fig 3E) reaction states, patients showed significant (p<0.02, p<0.03) up regulation of the CD4^+^CCR6^+^ population as compared to the respective stable forms. Moreover, this subset of cells produced IL-17. As reported earlier[27], the present study also confirmed that CD4^+^IL-17^+^CCR6^+^ effector T cells were significantly (p<0.03) higher in stable BT than LL patients (Fig 3B and 3F). However, as noted above, the two reactions showed differences in subtypes of IL-17^+^ as well as IL-21^+^ cells. Both reaction types showed increase in the percentage of IL-17A^+^ cells (Fig 3B and 3F). Significantly higher IL-17A^+^ (p<0.03) but not IL-17F^+^ producing CCR6^+^Th17^+^ cells were seen in RR (Fig 3C) as compared to the non reactive counterpart. In ENL reactions however, both IL-17A^+^ and IL-17F^+^ cells showed increases as compared to stable LL (Fig 3F and 3G respectively). It is of interest that IL-21^+^effector cell populations was increased in RR as compared to BT patients (Fig 3D, p<0.01) whereas no differences were noted between ENL and LL patients (Fig 3H).

### p-STAT3^+^ IL-17 ^+^cells in leprosy reactions

**Fig 4:**
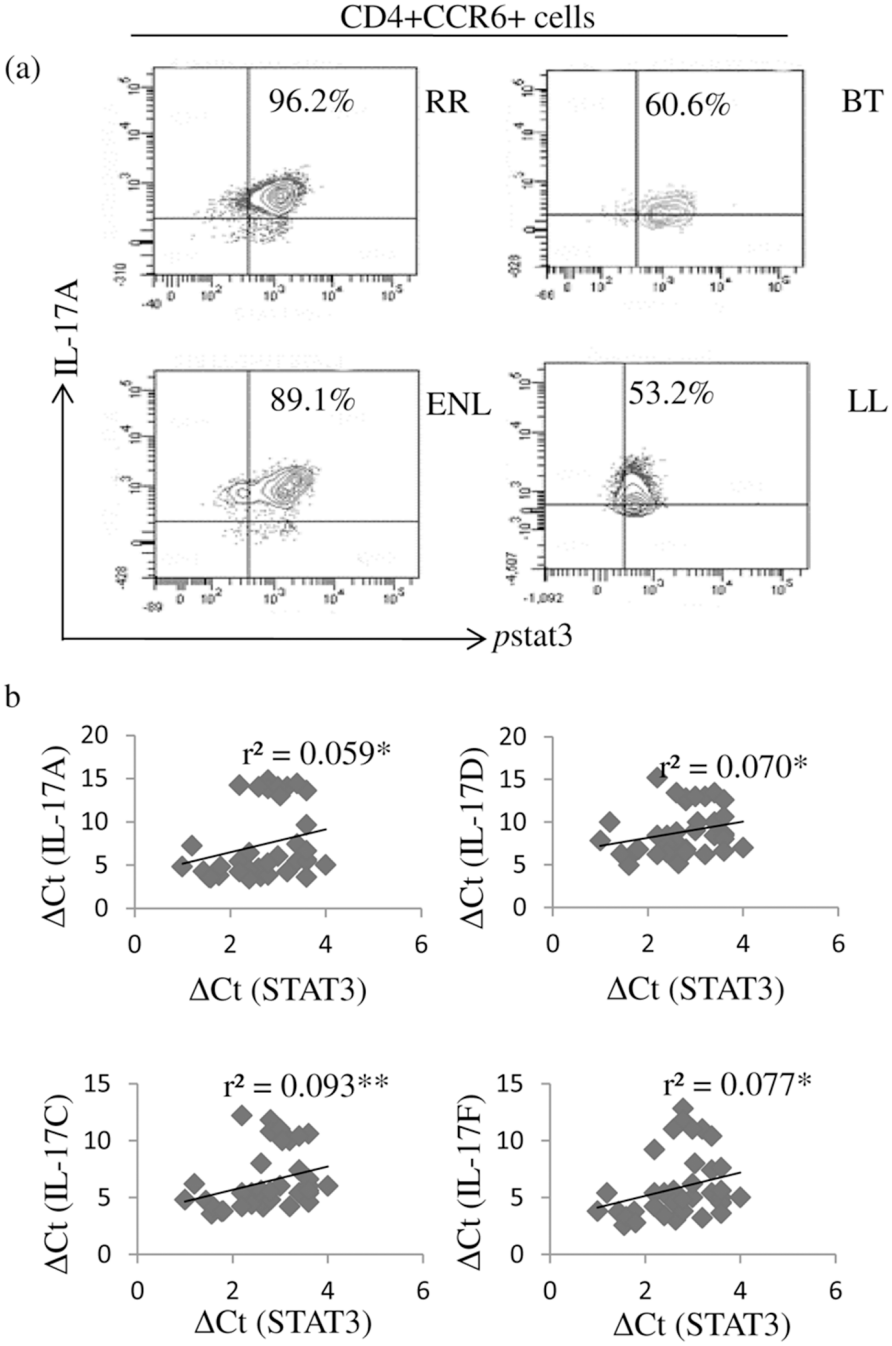
Increase in pSTAT3+IL17+ cells in leprosy reactions patients. Flow cytometry analysis of antigen stimulated 48 h culture of PBMC shows increase in pSTAT3 bearing CCR6^+^ cells with intracellular IL-17A in both RR and ENL subjects as compared to matching stable leprosy (a) Representative dot plots from each of RR, BT and ENL, LL leprosy patients (see Materials and Methods). (b) Scatter plots of combined qPCR data from both reaction patients RR and ENL, showing significant positive correlation of *STAT3* expression (ΔCT) with *IL17A*, *IL17D*, *IL17C* and *IL17F*. Correlation coefficient (r^2^) was calculated by Spearman non-parametric correlation test, p< 0.05 was considered statistically significant. p<0.01 = *, p<0.001 = **, Abbreviations as in Fig 1.

STAT3 expression by qPCR showed low / non significant increase in RR and ENL as compared to their respective stable forms of BT and LL groups in the antigen stimulated PBMC cultures (Fig 1). We further investigated the requirement for phosphorylation of STAT3 in CCR6^+^ IL-17A^+^ cells using flowcytometry in 3 of each leprosy patients. Fig 4A shows higher percentage of pSTAT3^+^IL-17^+^ cells in patients undergoing leprosy reactions as compared to non reaction patients. Moreover, co-expression of STAT3 and IL-17 isomers as indicated by qPCR also showed significant correlation (Fig 4B). (IL-17A; r^2^ = 0.059, p<0.01, IL-17D; r^2^ = 0.07, p<0.01, IL-17C; r^2^ = 0.093, p<0.001, IL-17F; r^2^ = 0.077, p<0.01)

### CD4^+^CD25^+^ FOXP3^+^ T cells

**Fig 5:**
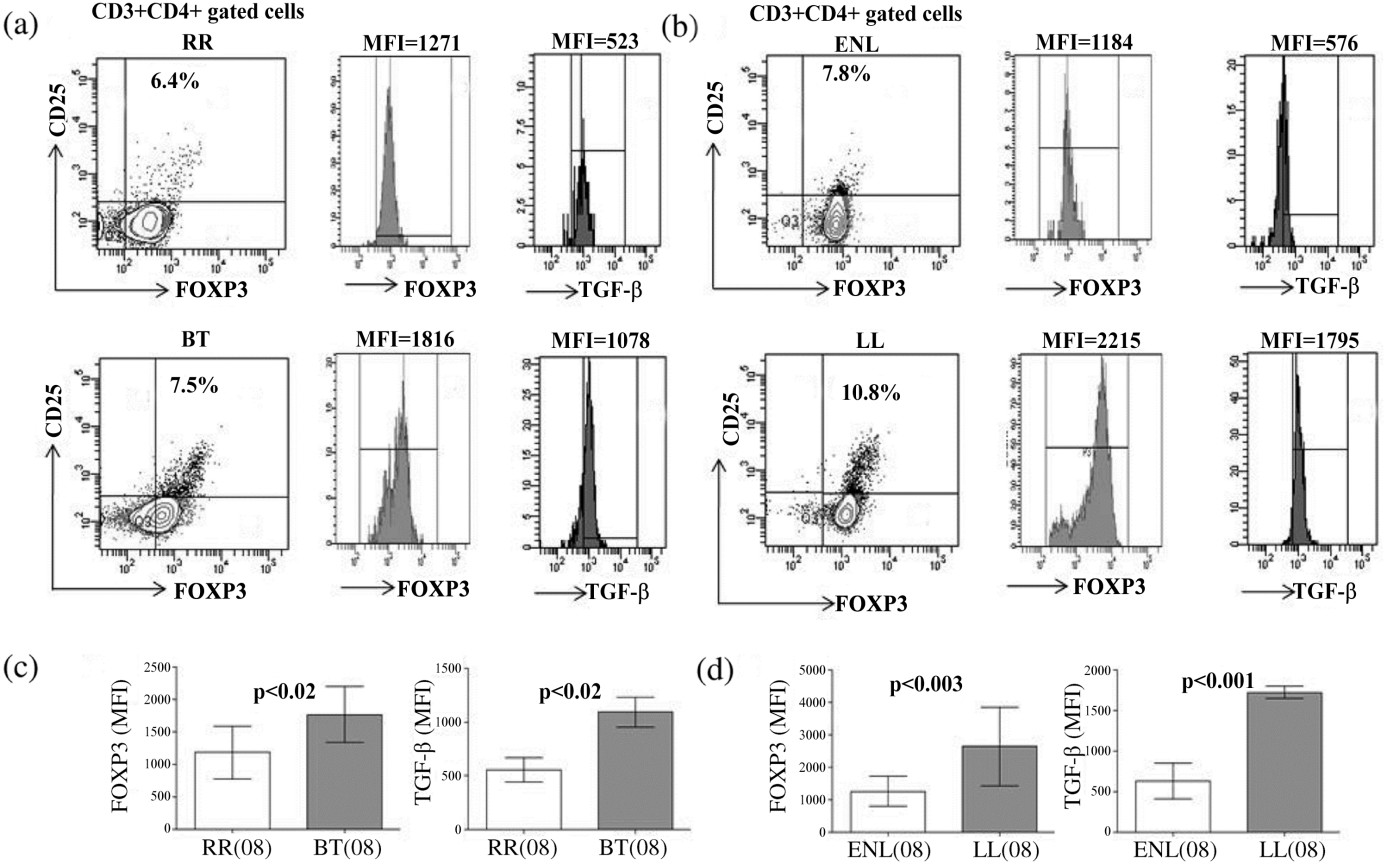
Reduction of FOXP3 and TGF-β in T cells of leprosy reaction patients. Flowcytometry analysis demonstrates reduction in percentage of CD3^+^CD4^+^gated CD25^+^FOXP3^+^ cells (see Materials and Methods). Dot plots show percentage of CD25^+^FOXP3^+^ cells in representative patients with RR, ENL as compared to stable BT, LL patients respectively. Histogram shows MFI (mean florescent intensity) of nuclear FOXP3 and intracellular TGF-β in each of RR and ENL as compared to respective BT and LL (Figs a, b). Bar diagram shows MFI of nuclear FOXP3 and TGF-β (mean±SD) in RR, ENL as compared to BT, LL leprosy groups respectively (Figs b, c). Data were analyzed using two tailed Mann-Whitney, p< 0.05 was considered as statistically significant. (), Parenthesis shows number of subjects tested. Abbreviations as Fig 1.

The role of FOXP3^+^ regulatory T cells (Treg) during leprosy reactions was investigated using both qPCR and flow cytometry analysis of *ex vivo* stimulated PBMC from both stable and matching leprosy reaction groups (Figs 1, 5a and 5b). In confirmation of our earlier studies [25], Tregs were increased in LL as compared to BT and produced TGF-β. RR and ENL patients however showed a decrease in Treg cells (p<0.02 to p<0.003) as compared to the nonreactive disease (Fig 5c and 5d). This was further confirmed by qPCR studies (Fig 1), where 5.5 fold increase in FOXP3 gene expression (p<0.001) was observed in stimulated PBMC from the RR and ENL group (p<0.008) as compared to patients with stable disease.

### Role of TGF-β and IL 6

We investigated levels of the inhibitory cytokine TGF-β using flow cytometry. Intracellular TGF-β was evaluated in CD4+CD25+FOXP3+Treg cells (Fig 5a and 5b). MFI (mean florescence intensity) showed significant (p<0.02) down regulation of TGF-β (Fig 5c) in patients showing both reactions states. RR patients showed significantly reduced MFI for TGF-β (p<0.02) as compared to BT as did ENL patients in comparison to stable LL patients (p<0.001, Fig 5d). However TGF-β expression by qPCR showed no change in RR and ENL as compared to the matching stable leprosy groups (Fig 1 and Table 3) which may be related to the difference in time kinetics in the PBMC cultures for expression of the genes as compared to the intracellular cytokine production.

**Fig 6:**
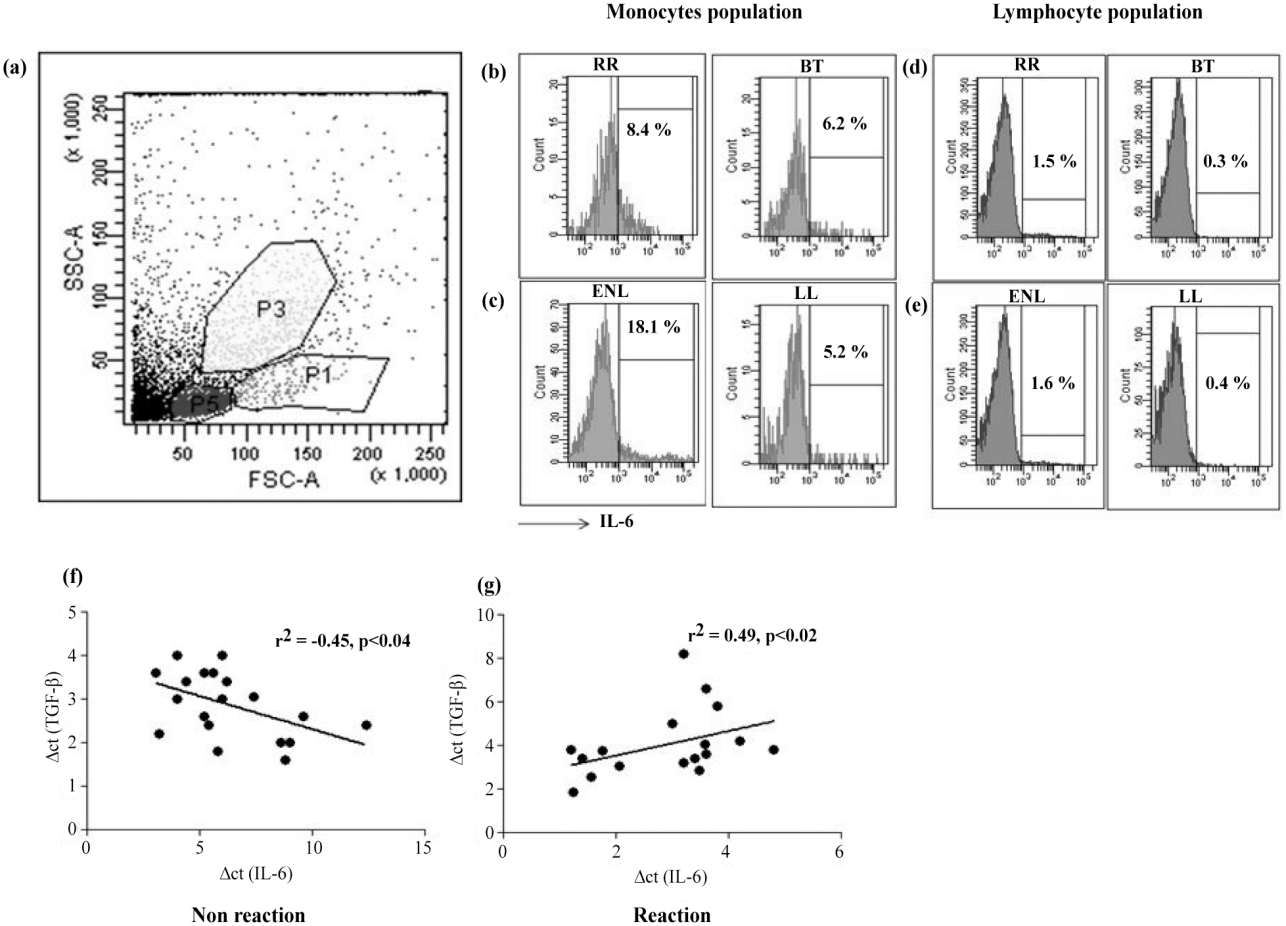
IL-6 increase in leprosy reactions is associated with monocyte population in antigen stimulated PBMC cultures of patients. Fig 6(a) shows monocytes gating strategy in flowcytometry. Histogram represents percentage of IL-6^+^ population in monocyte and lymphocyte populations in one each of (b, d) RR, BT and (c,e) ENL, LL respectively. Scattergrams shows pooled qPCR data where *TGF-β* and *IL-6* gene expression was undertaken on antigen stimulated PBMC of patients of (f) non-reaction stable (BT, LL) and (g) leprosy reaction (RR, ENL) patients. Correlation coefficient (r^2^) was calculated by Spearman nonparametric correlation test, p< 0.05 was considered statistically significant. Abbreviations as in Fig 1.

Recent reports [21,23,24] support the idea that Th17 differentiation is controlled by the opposing roles of TGF-β and IL-6. In contrast, we found that TGF-β, IL-6 expression by qPCR showed up-regulation during both reactions (p<0.01–0.005) as compared to the stable forms (Fig 1). We then investigated the nature of cells producing IL-6 in four patients of each leprosy group. Lymphocyte populations did not show any evidence of intracellular IL-6 (Fig 6a). Instead, flow analysis indicated that the cell populations gated for monocytes and granulocytes predominated. (Fig 6b and 6c). Both types of leprosy reactions showed increases in percentages of IL-6^+^ monocytes (p<0.02) as compared to stable leprosy, whereas the IL-6^+^ granulocyte population showed no differences in the reactive and non reactive groups (Table 4).

**Table 4:** Mean percentage ± SD of IL-6^+^ cells in gated populations of monocytes, granulocytes and lymphocytes in antigen stimulated PBMC of four patients each of stable tuberculoid (BT), lepromatous (LL) and corresponding leprosy reactions RR and ENL respectively studied by flowcytometry as depicted in Fig 6.

Gated cell populations	IL-6^+^ cells (Mean % ± SD) in patient groups			
	RR	BT	ENL	LL
Monocytes	8.6±1.0*	4.4±0.6	18.8±0.6*	4.1±0.2
Granulocytes	23.5±6.6	17.4±0.8	15.5±0.8	15.6±0.5
Lymphocytes	1.7±0.3	1.1±0.3	1.6±0.4	0.7±0.4

*p< 0.04, by two tailed Mann Whitney test. P<0.05 was considered significant.

Patient classification and abbreviations as in Table 1

The opposing role of these two cytokines were analyzed using Spearman correlation coefficient by pooling data obtained in qPCR studies from the reaction groups as compared to non-reactive groups (Fig 6f and 6g). TGF-β and IL-6 revealed opposing effects with negative correlation (r^2^ = -0.45, p<0.04) in stable leprosy and a positive correlation in leprosy reactions (r^2^ = 0.49, p<0.02).

### Differential expression of genes in reversal and ENL reactions

**Fig 7:**
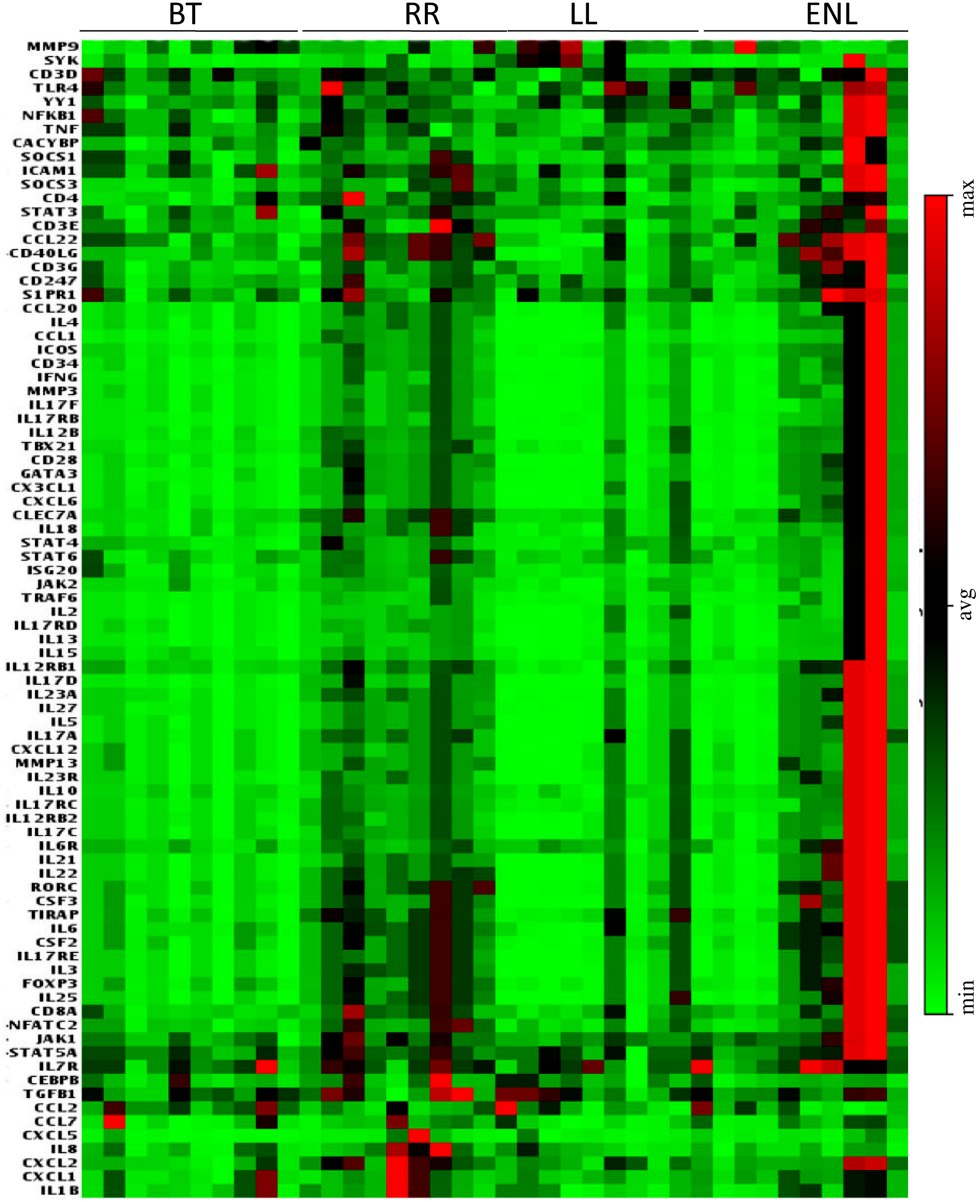
Heat map of customized PCR array showing differential expression of 84 genes in antigen stimulated PBMC of leprosy groups. **E**ach horizontal row represents the same gene product and each vertical row the same patient. The fluorescence range from high (red) to low (green) is indicated by the colored bar and reflects the degree of fluorescence intensity/gene expression. Heat map obtained from online free software S.A Bioscience (pcrdataanalysis.sabiosciences.com). In general, PBMC of patients in reactions show higher expression of several genes representing, cytokines, cytokine receptors, cheekiness and transcription factors as compared to non reaction patients (see also Table 5). Abbreviations as in Fig 1.

With a view to understanding the differences between the immunopathology/inflammation associated with the localized reversal and generalized ENL forms of reactions we analyzed the expression of 84 genes in qPCR assay (Fig 7 and Table 5). Heat map of gene expression as reported earlier [27], shows that stable BT subjects had higher expression of many genes as compared to anergic LL. Further increase in expression was observed in reaction subjects. Although there was individual variability, in general, RR subjects showed higher expression of genes expressing cell surface markers, cytokines, cytokine receptors, chemokines, signaling molecules and transcription factors as compared to ENL/Type 2 reactions. Table 5 provides data on the stastical significance of the fold changes in gene expression in patients with these two reaction types

**Table 5:** Expression of surface molecules, cytokines, signaling molecules, chemokines, receptors and transcription factors evaluated by qPCR where fold change between stable and reaction groups showed significant differences in RR and ENL.

	RR	ENL		RR	ENL		RR	ENL
Surface Molecules			Cytokines			Signaling Molecules, Transcriptional Factors		
CD247	ns	ns	CSF2	0.001	0.02	CEBPB	ns	0.03
CD28	0.0001	0.03	CSF3	0.001	0.02	CLEC7A	0.0001	ns
CD34	0.0001	ns	IFN-γ	0.001	0.05	S1PR1	ns	ns
CD3D	ns	ns	IL10	0.001	ns	FOXP3	0.001	0.03
CD3E	ns	0.01	IL12B	0.0001	ns	GATA3	0.0002	0.04
CD3G	ns	0.02	IL13	0.001	ns	JAK1	0.02	0.03
CD4	0.01	ns	IL15	0.001	ns	JAK2	0.01	ns
CD40LG	0.001	0.006	IL17A	0.0001	0.002	MMP13	0.001	ns
CD8A	0.001	ns	IL17C	0.001	0.005	MMP3	0.0003	ns
ICAM1	ns	ns	IL17D	0.001	0.04	MMP9	ns	ns
ICOS	0.0001	0.04	IL17F	0.001	0.001	NFATC2	0.005	ns
ISG20	ns	ns	IL18	0.002	ns	NFKB1	ns	ns
Chemokines			IL1β	ns	ns	RORC	0.0001	0.02
CCL1	0.0001	ns	IL2	0.0001	ns	SOCS1	ns	ns
CCL2	ns	ns	IL21	0.0001	0.04	SOCS3	0.01	ns
CCL20	0.0001	ns	IL22	0.0001	0.04	STAT3	ns	ns
CCL22	0.001	0.005	IL23A	0.0001	0.04	STAT4	0.01	ns
CCL7	ns	ns	IL25	0.0001	ns	STAT5A	ns	ns
CX3CL1	0.001	ns	IL27	0.0001	ns	STAT6	ns	ns
CXCL1	ns	ns	IL3	0.0001	ns	SYK	ns	ns
CXCL12	0.001	ns	IL4	0.0001	ns	TBX21	0.0001	ns
CXCL2	0.001	ns	IL5	0.0001	0.04	TIRAP	0.0001	ns
CXCL5	ns	ns	IL6	0.0001	0.02	TLR4	ns	ns
CXCL6	0.001	ns	TGFβ1	ns	ns	CACYBP	0.04	ns
IL8	ns	ns	TNF	ns	ns			
Cytokine Receptors								
IL12RB1	0.0008	0.04	IL7R	ns	ns	IL17RD	0.0001	ns
IL12RB2	0.001	ns	IL17RB	0.001	ns	IL17RE	0.0002	0.03
IL23R	0.0002	ns	IL17RC	0.0001	ns	IL6R	0.01	ns

p value calculated by Student’s t test. ns = non significant, Abbreviations as in legend to Table 1.

## Discussion

The present study was undertaken with a view to understanding the inflammation/immunopathology seen in patients undergoing episodes of leprosy reactions which are a cause of severe morbidity and nerve damage. Previous studies had shown that Th17 cells formed a third subset in leprosy and were seen in stable leprosy patients in the absence of Th1 and Th2 polarization[27]. Of interest was the finding that IL-17 and its isomers were up regulated in leprosy reactions as compared to non-reactive leprosy patients. Reaction patients showed higher expression of IL-17A and its isomers as well as associated cytokines IL-21, IL-22 which was further confirmed by ELISA in culture supernatants. Our results are in conformity with a recent study showing increased serum levels of IL-17F in reaction patients[28]. In conformity with our earlier reports antigen stimulated PBMC of both types of leprosy reactions also showed IL-17 to be present in CD4^+^CCR6^+^ cells, along with the signature transcription factor RORC and pSTAT3 [27]. However, it was of interest to note that there was a difference in the associated cytokines between the two reactions. Increase of IL-17F bearing CCR6^+^ cells, was seen in reversal reactions (RR, Type 1) and not in Erythema Nodosum Leprosum (ENL, Type 2) patients. In contrast, CCR6^+^ cells bearing IL-21 were increased in ENL but not in RR. It was not possible to determine whether these differences were substantive or related to time kinetics of the human immune responses as it is not possible to clinically determine the time of initiation of the reactional state.

The development of Th17 cells is dependent on several cytokines which may act singly or in synergy with other cytokines [30,34]. We next investigated IL-23A, IL-1β and IL-6 which have been implicated in the development and differentiation of Th17 cells [30,31,35,36]. Our previous studies on stable tuberculoid leprosy had indicated that IL-23 and IL23R but not IL-6 and its receptor (IL-6R) were associated with Th17 cell differentiation[27]. In the present study IL-6 was significantly increased in both types of leprosy reactions as compared to matching non reactive patients (p <0.01 and 0.001 in RR and ENL respectively) and correlated significantly with STAT3 gene expression (p < 0.03) indicative of the signaling requirements for Th17 cells. The source of IL-6 was found to lie in the monocyte, granulocyte population and the percentage of IL6^+^monocytes were increased in both types of leprosy reaction patients. That monocyte/macrophage family are not only the hosts for the intracellular pathogen *M*.*leprae* but also play a role in the immune responses of the leprosy disease has been shown earlier by us[37] and others[38]. IL-6 has been reported to be upregulated in autoimmune diseases [39].

Using flow cytometry, the present study confirmed the increase in inhibitory FOXP3^+^Treg cells and TGF-β^+^ cells in stable non reactive lepromatous leprosy patients[25,26] which may be related to the T cell unresponsiveness observed in this form of leprosy. Importantly, Tregs were reduced in lepromatous patients ENL. RR patients also showed relative increase as compared to matching stable BT patients. In both types of reactions, CD4^+^CD25^+^ Treg cells showed significant reduction in MFI of FOXP3 in antigen stimulated PBMC of (p<0.02, p<0.003) though qPCR showed an increase in its expression as compared to stable non reactive lepromatous leprosy patients. The variability in FOXP3 expression may be due to whole PBMC included in qPCR studies whereas individual Treg cells were studied by flowcytometry. Alternatively, the results may indicate the transient nature of FOXP3 expression [25,40]. The reduction in Treg cells may lower the inhibitory effects and thereby influence the enhanced Th17 activity observed in leprosy reactions. Thus leprosy reactions show an imbalance of Th17 cells and regulatory T cells tipping the balance towards inflammation. Such an imbalance has been reported in tuberculous pleural effusion[41], respiratory syncytial virus infection in children[12] and at an early stage of HIV infection where protective CD8^+^ cells were noted. In HIV+ patients better clinical status correlated with higher Th17 and lower Treg cells [13]. Such a perturbation was considered to contribute to the patho-physiology of autoimmune disease[10]. That Th17 cells may confer protection in tuberculosis by vaccine MVA85A was shown in a murine model[42].

The development and differentiation pathways of Th17 and Treg cells are influenced by various cytokines. TGF-β along with the pro-inflammatory cytokines IL-6, IL-21 and IL-1β drive naïve T cells towards Th17 differentiation [43,44] withIL23 playing a role in stabilizing this population[31]. TGF-β also drives cells towards FoxP3^+^ T regulatory cells and inhibits Th17 differentiation [9,19]. IL-6 appears to have a role in determining Th17/Treg balance as along with minimal concentrations of TGF-β it induces Th17 differentiation [21,24,45,46] and inhibits Treg differentiation. IL-23[47], CCL20[48] required for maintenance of Th17 cells and IL23R also showed enhanced expression in leprosy reactions as compared to the respective non reactive groups. IL-21 showed a mixed pattern in flow cytometry, as it was linked to CD4+ cells in RR but not in ENL. When CCR6+ cells were examined IL-21 showed the opposite pattern being associated in ENL and not RR. Taken together, our studies indicate that Th17 cell induction in leprosy reactions is related predominantly to over production of IL-6[49]combined with low TGF-β.

84 genes defining cell markers, cytokines, chemokines, receptors and signaling molecules were investigated in all 4 clinical groups using qPCR and antigen stimulated PBMC. We confirmed our earlier observations[27] that stable forms of tuberculoid leprosy showed higher levels of gene expression as compared to anergic lepromatous leprosy. Importantly, the number of genes showing increased expression was higher in RR as compared to ENL subjects. There was some individual variation which may be attributable to the stage of reaction which is difficult to ascertain clinically. The increase in gene expression of cytokines and other markers selectively in Type 1 reactions may be related to the presence of cell mediated immune reactions in tuberculoid leprosy which is the leprosy type of RR patients as compared to ENL who have lepromatous leprosy with negligible T cell functions.

In conclusion, our study shows that the equilibrium between Th17 and Treg cells is disturbed in leprosy reactions with an increase in Th17 cells and reduction in Treg cells. This imbalance is mediated by cytokines as there is concurrent reduction in TGF-β and increase in monocyte derived IL-6. Enhanced Th17 cell activity would explain the inflammation and immuno-pathology associated with leprosy reactions. Furthermore, the increased gene expression of CCL20 and CCL22chemokines in reaction states may facilitate Th17 cell migration to inflammatory sites[50,51]. Thus our studies indicate a shift in our understanding of the immunological features that mediate and regulate leprosy reactions with a new paradigm that is beyond the conventional Th1 and Th2 subsets of T cells.

## Supporting Information

S1 Table Gene accession numbers. (DOCX)
