# Supplementary material for: Leprosy Reactions Show Increased Th17 Cell Activity and Reduced FOXP3+ Tregs with Concomitant Decrease in TGF-β and Increase in IL-6
Source: PLoS Negl Trop Dis. 2016 Apr 1;10(4):e0004592. doi: 10.1371/journal.pntd.0004592 (PMC4818038; doi:10.1371/journal.pntd.0004592)
Supplement: S1 Table — (DOCX) [file pntd.0004592.s001.docx]

**S1 Table:** Gene accession numbers.

| **Symbol** | **Unigene** | **Symbol** | **Unigene** | **Symbol** | **Unigene** | **Symbol** | **Unigene** |
| --- | --- | --- | --- | --- | --- | --- | --- |
| CACYBP | Hs.508524 | CXCL1 | Hs.789 | IL17RB | Hs.654970 | JAK1 | Hs.207538 |
| CCL1 | Hs.72918 | CXCL12 | Hs.522891 | IL17RC | Hs.129959 | JAK2 | Hs.656213 |
| CCL2 | Hs.303649 | CXCL2 | Hs.590921 | IL17RD | Hs.150725 | MMP13 | Hs.2936 |
| CCL20 | Hs.75498 | CXCL5 | Hs.89714 | IL17RE | Hs.390823 | MMP3 | Hs.375129 |
| CCL22 | Hs.534347 | CXCL6 | Hs.164021 | IL18 | Hs.83077 | MMP9 | Hs.297413 |
| CCL7 | Hs.251526 | S1PR1 | Hs.154210 | IL1B | Hs.126256 | NFATC2 | Hs.713650 |
| CD247 | Hs.156445 | FOXP3 | Hs.247700 | IL2 | Hs.89679 | NFKB1 | Hs.654408 |
| CD28 | Hs.591629 | GATA3 | Hs.524134 | IL21 | Hs.567559 | RORC | Hs.256022 |
| CD34 | Hs.374990 | ICAM1 | Hs.643447 | IL22 | Hs.287369 | SOCS1 | Hs.50640 |
| CD3D | Hs.504048 | ICOS | Hs.56247 | IL23A | Hs.98309 | SOCS3 | Hs.527973 |
| CD3E | Hs.3003 | IFNG | Hs.856 | IL23R | Hs.677426 | STAT3 | Hs.463059 |
| CD3G | Hs.2259 | IL10 | Hs.193717 | IL25 | Hs.302036 | STAT4 | Hs.80642 |
| CD4 | Hs.631659 | IL12B | Hs.674 | IL27 | Hs.528111 | STAT5A | Hs.437058 |
| CD40LG | Hs.592244 | IL12RB1 | Hs.567294 | IL3 | Hs.694 | STAT6 | Hs.524518 |
| CD8A | Hs.85258 | IL12RB2 | Hs.479347 | IL4 | Hs.73917 | SYK | Hs.371720 |
| CEBPB | Hs.517106 | IL13 | Hs.845 | IL5 | Hs.2247 | TBX21 | Hs.272409 |
| CLEC7A | Hs.143929 | IL15 | Hs.654378 | IL6 | Hs.654458 | TGFB1 | Hs.645227 |
| CSF2 | Hs.1349 | IL17A | Hs.41724 | IL6R | Hs.709210 | TIRAP | Hs.537126 |
| CSF3 | Hs.2233 | IL17C | Hs.278911 | IL7R | Hs.591742 | TLR4 | Hs.174312 |
| CX3CL1 | Hs.531668 | IL17D | Hs.655142 | IL8 | Hs.624 | TNF | Hs.241570 |
| YY1 | Hs.388927 | IL17F | Hs.272295 | ISG20 | Hs.459265 | TRAF6 | Hs.591983 |
| B2M | Hs.534255 | HPRT1 | Hs.412707 | RPL13A | Hs.523185 | GAPDH | Hs.592355 |
| ACTB | Hs.520640 |  |  |  |  |  |  |
